# Bakdrive: identifying a minimum set of bacterial species driving interactions across multiple microbial communities

**DOI:** 10.1093/bioinformatics/btad236

**Published:** 2023-06-30

**Authors:** Qi Wang, Michael Nute, Todd J Treangen

**Affiliations:** Systems, Synthetic, and Physical Biology (SSPB) Graduate Program, Rice University, Houston, TX 77005, United States; Department of Computer Science, Rice University, Houston, TX 77005, United States; Department of Computer Science, Rice University, Houston, TX 77005, United States

## Abstract

**Motivation:**

Interactions among microbes within microbial communities have been shown to play crucial roles in human health. In spite of recent progress, low-level knowledge of bacteria driving microbial interactions within microbiomes remains unknown, limiting our ability to fully decipher and control microbial communities.

**Results:**

We present a novel approach for identifying species driving interactions within microbiomes. Bakdrive infers ecological networks of given metagenomic sequencing samples and identifies minimum sets of driver species (MDS) using control theory. Bakdrive has three key innovations in this space: (i) it leverages inherent information from metagenomic sequencing samples to identify driver species, (ii) it explicitly takes host-specific variation into consideration, and (iii) it does not require a known ecological network. In extensive simulated data, we demonstrate identifying driver species identified from healthy donor samples and introducing them to the disease samples, we can restore the gut microbiome in recurrent *Clostridioides difficile* (rCDI) infection patients to a healthy state. We also applied Bakdrive to two real datasets, rCDI and Crohn's disease patients, uncovering driver species consistent with previous work. Bakdrive represents a novel approach for capturing microbial interactions.

**Availability and implementation:**

Bakdrive is open-source and available at: https://gitlab.com/treangenlab/bakdrive.

## 1. Introduction

Host-associated microbes exist as part of complex and dynamic ecosystems which have a profound impact on human. Disruption of microbial communities is frequently observed after perturbations, including after antibiotic and non-antibiotic drug administration (Maier et al. 2018; Palleja et al. 2018; Gibbons 2020), and in many diseases, including recurrent *Clostridioides difficile* infection (rCDI) and inflammatory bowel disease (IBD) (Menon et al. 2018; Hirano and Takemoto 2019; Wu and Savidge 2020). Furthermore, several previous studies have investigated the specific ecological roles of human-associated microbes in synthetic experiments with the goal of enabling the design of beneficial microbial communities (Venturelli et al. 2018). Therefore, the restoration of human gut microbiota to healthy states through microbiome-based therapies, such as fecal microbiota transplantation (FMT), is a promising future therapeutic modality for improving human health (Ooijevaar et al. 2019; Wong and Levy 2019).

FMT is the most successful treatment to date in restoring microbial communities and is a clinically approved therapy for rCDI (Wang et al. 2019). During FMT, fecal material from a healthy donor is introduced into a recipient with dysbiosis with the hope that the newly transplanted microorganisms will engraft in the recipient and jump start the restoration to a healthier community state. However, the FMT procedure is a high complexity and non-targeted intervention, and there is an inherent risk that the FMT could have negative health consequences (Davido et al. 2018; Merrick et al. 2020). Furthermore, several key questions surrounding FMT remain unanswered. Which species are most important for restoring dysbiosis? How do species interact with each other during the FMT and in the new host environment? Accurate identification of keystone species in such situations remains an open research question (Banerjee et al. 2018; Röttjers and Faust 2019). If the mechanisms driving the underlying biology were better understood, a community could be developed that is simpler, standardized, and that could be engrafted in the same procedure without the risk associated with standard FMT. It would also serve a more general purpose of improving microbiome-targeting therapeutics and pushing microbiome research closer to clinical application (Lemon et al. 2012; Kashyap et al. 2017). The particular challenge in developing such a community is in identifying both the necessary and sufficient bacterial species that should comprise it. There is no obvious computational approach for identifying a sub-community that would serve as such a functional proxy for an actual FMT.

In our study, we apply a minimum dominating set (MDS) approach to find a set of driver species for the community. In the MDS method applied to a generic undirected graph, a set of driver nodes is one to which all the other nodes in the network directly connect (Molnár et al. 2013). It assumes that one driver node can control all the direct connected nodes. This concept has been applied to biology in studies demonstrating that an MDS can be used to find driver genes or proteins with meaningful biological functions (Nacher and Akutsu 2014; Akutsu 2016; Kanhaiya et al. 2017), for example.

Here, we apply the same concept to a network representing bacterial interactions in order to identify a set of driver species within a healthy human gut microbiome. The set of species that are identified are then used in an *in silico* experiment simulating the effects of engrafting that community in a host affected by *C.difficile*. Specifically, the relative abundance of the host microbiome is simulated to additional timepoints post-engraftment, which is itself a proxy for host recovery in as much as it reflects a reduction in the abundance of *C.difficile* specifically. We show that a set of driver species chosen using this MDS approach, after simulated engraftment, results in host “recovery” in simulation and that its predicted effects are comparable to that of FMT *in vivo*. We further show that this method is robust to assumptions about the specific bacterial interaction network, and that its performance improves as it incorporates data from many patients.

### 1.1 Previous work

Applying controllability analysis to shift microbiome states, which are characterized by microbial abundance profiles, is a field still in its infancy (Kuntal et al. 2019). Nevertheless, three recent publications have attempted to tackle this open research question (Table 1). Gibson et al. (2016) was one of the first attempts to explore the concept, identifying “strongly interacting species” (i.e. driver-species) and they suggest that a small number of these species can potentially shift metagenomic samples to desired states. This analysis is strictly based on randomly generated assumptions for the interaction network. Nevertheless, they set up a framework for testing the effect of perturbations using the *Generalized Lotka-Voltera* simulation model (Hernández-Bermejo and Fairén 1997), which has become a common practice to evaluate the controllability of a given microbiome community (van den Berg et al. 2022).

**Table 1.** Existing methods for identification of driver species in microbial communities.

	Identifies driver species	Handles multiple samples	Requires known network	Infers ecological networks
Gibson et al.	✓		✓	
Angulo et al.	✓		✓	
Xiao et al.	✓		✓	
Bakdrive	✓	✓		✓

More recently, Angulo et al. (2019) develop an algorithm to compute a minimum set of drivers given a parameterized microbial interaction network. They show that the minimum set of driver species has the ability to control microbial communities in the simulation environment under the assumed interaction networks (Angulo et al. 2019).

Finally, Xiao et al. construct a model and simulation framework specifically to represent the FMT process. One particular advancement over the previous simulation model is that the simulation conditions are specific to a “patient.” Specifically, it assumes there is a single, parameterized global interaction network for all patients (Bashan et al. 2016). However, for a given patient, not all organisms in the global network may be present. Thus, the interaction network of that patient is taken as the induced subgraph of the global network. The resulting diversity of simulation conditions stresses the assumptions of the previous simulation model and introduces patient-specific variability.

A key challenge in all three of these works noted by the authors is the difficulty of properly estimating the global interaction network parameters (Dohlman and Shen 2019; Lv et al. 2019). This estimation for a particular host or phenotype is typically a painstaking endeavor, with many samples required to identify the strength of interactions. Another recently released software tool, called MICOM, took a very different approach, using a flux balance analysis to derive an interaction network from microbial genomes. It uses a broad metabolic model of the human gut microbiome and genome-wide metabolic profiles for individual microbes to impute the pairwise promotion/inhibition effects and construct the interaction network in that way (Diener et al. 2020). The effect of this is that a credible interaction network can be constructed *de novo* for each individual patient based on their community’s metagenomic composition, which would be a substantial departure from the global ecological network inherent in previous work.

An advantage of using patient-specific interaction networks, aside from sidestepping a tedious assumption, is that the range of networks across a group of patients contains information about the variance of this feature within the population, which could be useful. For the purpose of identifying a common set of driver species for the group of samples, an algorithm that can operate with multiple networks is needed. Previously, Nacher et al. (2019) have discussed this particular problem setup, where multiple different networks have no underlying connection to one another and can be controlled by a common set of driver nodes. This set of networks is represented as a multilayer network where each layer of network is independent of each other. Nacher et al. present a heuristic multilayer MDS (called an MDSM) method for estimating driver nodes, and investigate their controllability of multilayer networks.

## 2. Materials and methods

### 2.1 Assumption-free MDS computation

Here, we present a method called Bakdrive that combines the MICOM sample-specific interaction networks with the MDSM approach to find a set of driver species. Specifically, MICOM is a published software tool that produces a sample-specific bacterial interaction network based on the specific bacteria present and their relative abundances (Diener et al. 2020). Additionally, MICOM was leveraged in previous microbiome study to predict bacteria interaction networks (Basile et al. 2020). MICOM is a widely accepted genome-scale metabolic modeling tool that can provide valuable insights in microbiome characterization (Ankrah et al. 2021), although notably MICOM was developed primarily for human microbiomes and thus a different sample-specific interaction network may be more suitable for other applications (e.g. environmental microbiomes). Regardless of the source, Bakdrive takes the relative abundance from *multiple* samples as its input (or alternatively, the interaction network from those samples) and produces a set of driver species as its output. The algorithm operates in two main steps:

1. The bacterial interaction networks for each sample is inferred from MICOM (unless provided explicitly) are combined into a single multilayer network.
2. The multilayer MDS algorithm from Nacher et al. (2019) (herein, simply the MDSM algorithm) is applied to compute a set of driver nodes for the group.

This two-part method contains some important innovations over previous methods. For one, our method does not depend pivotally on the assumption of a single global interaction network which, while not obviously problematic, is necessarily a major simplifying assumption for both computing the MDS and simulating outcomes. Furthermore, the interaction network is computed *de novo* for each sample and thus does not need to be provided as an assumption *a priori* (Table 1). One property which we demonstrate in the experiments that follow is that the effectiveness of the driver species set is related to the number of samples used as input. In other words, each sample is, in essence, a data point that the algorithm uses to find a representative driver set, with more data leading to a better outcome.

In the experiments described below, we test the controllability of driver species identified by Bakdrive using the rCDI FMT process simulation framework developed by Xiao et al. By varying the number of samples contributing to the multi-layer interaction network (or in other words by varying the number of layers), we show that the ability of the MDS to control the community “is directly related to the size and thus diversity of the input set.” We then test the robustness of identified driver species by increasing the variations of given multilayer networks: we randomly rewire the interaction networks for each sample by adding and removing edges, then re-run the experiments. Even with this random noise introduced, the MDS computed in this manner shows only modestly reduced effectiveness at controlling the community and remediating the rCDI condition. We then compare the predictions of this model to the changes observed in an actual FMT study on rCDI patients, and we show that the simulated changes induced by the Bakdrive MDS transplantation (MDST) closely resemble actual community changes in the FMT recipients. Finally, Bakdrive shows comparable efficacy to the method of Xiao et al., with the advantage that Bakdrive is not specifically designed for a single target pathogen.

### 2.2 Bakdrive implementation

We implemented the Bakdrive pipeline to contain three main modules: (i) bacterial interactions inference (MICOM interaction module), (ii) driver species identification (driver module), and (iii) FMT/MDST simulation for validation experiments. For FMT/MDST simulation, it contains three different simulations. **FMT_donor** module simulates the FMT process: adding donor samples directly to diseased samples, inferring bacteria interactions and simulating the after-FMT species abundance changes following the GLV model. **FMT_driver** module simulates the MDST process: adding equal amounts of driver species to the diseased samples, inferring bacteria interactions of after-MDST samples and simulating the after-MDST species abundances changes. **FMT_only** takes the inferred bacteria interactions of after-FMT/MDST samples and simulates the abundances changes directly. The source code is available at: https://gitlab.com/treangenlab/bakdrive.

### 2.3 Simulation of rCDI FMT with universal dynamics

**Figure 1.**
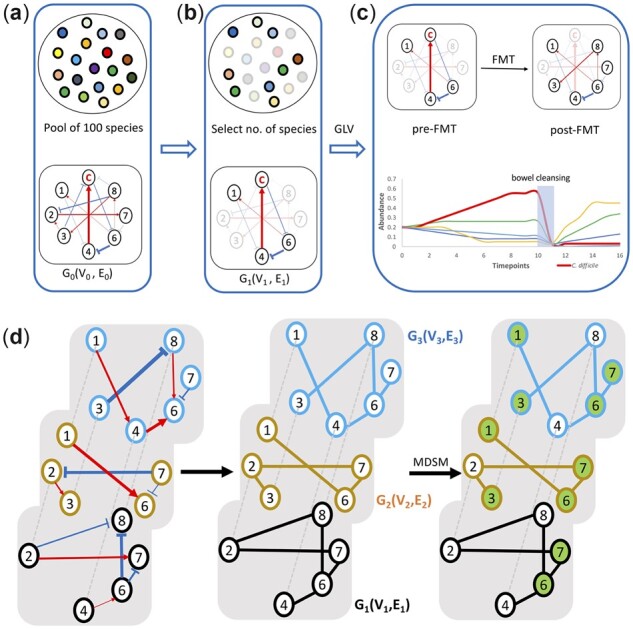
FMT process simulation and driver species identification. (a) The ecological network *G*_0_ of a metacommunity with 100 species. Red/blue arrows represent positive/negative relationships respectively. The widths of arrows reflect interaction strengths. (b) A subset of species and their corresponding interaction networks *G*_1_ are randomly selected from the pool of 100 species. (c) Simulation of species abundance over time. The thick red line represents the abundance of C. difficile. The FMT process simulation includes the pre-FMT bowel cleansing process, which leads to sudden drops of species abundance in pre-FMT samples. (d) Driver species identification. Directed and weighted bacteria interaction networks are converted to an undirected, unweighted multilayer network where layers are unconnected. Here, the multilayer network consists of three layers: $G_{1}$($V_{1},\;E_{1})$, $G_{2}$($V_{2},\;E_{2})$, $G_{3}$($V_{3},\;E_{3})$. Green nodes represent driver nodes found using the MDSM algorithm. All non-driver nodes directly connect to at least one driver node.

Simulation experiments are depicted in Fig. 1 and closely follows the procedures in Xiao et al. (2020). We follow the modeling procedures from Xiao et al. to simulate 1000 pairs of healthy (donor) and pre-FMT diseased metagenomic samples. Each simulated metagenomic sample is a random sampling from a metacommunity of 100 species (Fig. 1a–c). The initial abundance of sampled bacteria is 0.2. The time-dependent bacteria abundances change following the Generalized Lotka-Volterra (GLV) model with an interaction matrix A and an intrinsic growth rate vector r as inputs. The simulation process is built on the assumption that gut microbiomes have strong universal dynamics for healthy adults (Xiao et al. 2020; Bashan et al. 2016). Thus, all initial simulated microbial communities have the same bacteria interaction network A and initial growth rate r but with different species compositions.

Our main deviation from Xiao et al. is the choice of driver species, which uses Bakdrive. After simulation, we randomly select N= 1, 5, 10, 20, 50, and 100 samples from the 1000 donor samples to build an N-layer network, and apply the MDSM approach to find driver nodes from the N-layer network (Fig. 1d). After that, we introduce the driver species to the 1000 simulated diseased samples, while excluding driver species that have already existed in the diseased samples (the most effective FMT method in Xiao et al.). The exclusion of species already present is a potential limitation of the method as the quantity of that particular species may affect the recovery post engraftment.

To quantify the efficacy of MDST, we borrow the concept of recovery degree, μ = $\frac{x^{d}-x^{p}}{x^{d}-x^{h}}$, where $x^{h}$, $x^{d},$ and $x^{p}$ are the abundances of *C.difficile* in healthy, diseased and after-MDST states. We repeat each experiment of the N-layer network three times.

### 2.4 Simulation of rCDI FMT with host-dependent dynamics

To test the robustness of driver nodes in suppressing pathogens, we alternate interaction networks of each simulated metagenomic sample while preserving the degree distribution of individual nodes. This practice is commonly used in complex network study. We randomly select two edges ${\{v}_{i},\;v_{j}\}$ and ${\{v}_{h},\;v_{k}\}$ from the central graph $G_{0}$($V_{0}$, $E_{0}$). Meanwhile, ${\{v}_{i},\;v_{k}\}\notin E_{0}$ and ${\{v}_{h},\;v_{j}\}\notin E_{0}$. We add the interaction strengths and directions of ${\{v}_{i},\;v_{j}\}$ and ${\{v}_{h},\;v_{k}\}$ to ${\{v}_{i},\;v_{k}\}$ and ${\{v}_{h},\;v_{j}\}$, respectively, then delete ${\{v}_{i},\;v_{j}\}$ and ${\{v}_{h},\;v_{k}\}$ from the graph. We repeat this step 16 times. In this way, the degree of each node remains the same. After this the simulation experiments are repeate*d*.

### 2.5 MDSM algorithm

Before applying the MDSM algorithm, we need to convert a set of weighted, directed bacteria interaction networks into a multilayer network, where each layer is an undirected and unweighted bacterial interaction network $G_{k}$($V_{k}$, $E_{k}$) of a metagenomic sample k. Here, we simply consider the set of individual interaction networks as unconnected layers in a multilayer network, which it can then be used as an input to MDSM.

### 2.6 Real rCDI metagenomic data analysis

The rCDI metagenomic data includes 26 donor samples from 7 donors, 19 patient samples and their corresponding after-FMT samples. Among 19 patients, 12 fully recovered after receiving a single dose of FMT, while the remaining 7 patients needed a second round of FMT. For simplicity, we focus on analyzing the FMT results of the 12 patients with single successful FMT. The raw sequencing of rCDI is available at BioProject PRJNA454892. The sequences are classified at species level using Kraken with the full database (Wood and Salzberg 2014).

To identify driver species from real metagenomic data, we first construct bacteria interaction networks of individual metagenomic samples. The newly developed software MICOM provides a function of inferring bacteria interactions and species growth rate through flux balance analysis (FBA) (Diener et al. 2020). Before FBA, MICOM matches species in each sample with their genome-scale metabolic models by name. In this work, AGORA_1_03_With_Mucins is used as the model database, which contains 818 reconstructed models (Magnúsdóttir et al. 2017). If a species has multiple strains’ metabolic models in the database, we will randomly pick one as the representative model of the species. While conducting FBA, western diet is used as the default medium for growth simulation.

### 2.7 Driver species transplantation simulation of real rCDI data

Starting from estimating the absolute biomass of each species in each sample, we multiply the relative abundances of each species with estimated total human gut microbiota biomass. On average, the overall weight of human gut microbiota is 200 g (Sender et al. 2016). To simulate driver species colonization of real data, we add equal amounts of driver species (default 40 g/species) to the clinical patient samples (Fig. 4). After that, MICOM infers bacteria interactions and intrinsic bacteria growth rates of the after MDST samples. It is worth mentioning that, as MICOM recommends, we divide predicted growth rates with estimated biomass in each sample, in this case, 200 g of bacteria. In terms of bacterial interactions, it is hard to predict interaction strength with exact 0 value using MICOM. Thus, we assume that low interaction strengths play little role in metagenomic dynamics. If the absolute value of an interaction strength is below a given threshold, the interaction strength is set to 0, that is, we assume there is no bacterial interaction between the two species. To choose a reasonable interaction strength threshold, we create a screen plot, where the x-axis is a list of absolute values of interaction strengths and the y-axis corresponds to $\alpha =\frac{\mathrm{total}\;\mathrm{number}\;\mathrm{of}\;\mathrm{species}}{\mathrm{number}\;\mathrm{of}\;\mathrm{drivers}}$. Based on empirical experiments, the threshold at the inflection point gives the best performance. In this study, we select 0.2 as the threshold for both *C.difficile* and IBD datasets (Supplementary Fig. S1). After adjusting growth rates and bacteria interaction strengths, we simulate the population dynamics following the GLV-based modeling framework proposed by Xiao et al. study (Xiao et al. 2020). The simulation stops at steady state or the last time point all the species have positive abundances. This step is performed by Bakdrive *fmt_driver* module.

### 2.8 Simulated and real rCDI FMT process comparison

To quantify the efficacies of simulated driver species transplantation, we calculate the percentage species in renormalized pre-FMT samples whose abundances move in the same directions in simulated and real post-FMT process. We also refer to this value as agreement. To be clear, we only examine species with metabolic models in AGORA database, that is, whose bacteria interactions can be estimated by MICOM. We first compute species abundance changes between patients’ pre-FMT samples and their post-FMT samples from the real data (Magnúsdóttir et al. 2017; Park et al. 2019). To compare the abundance shfits between real and simulated data, we renormalize the relative abundance of species in pre-FMT samples with species who have metabolic models in AGORA database. We then calculate species abundance changes between re-normalized pre-FMT samples and simulated post-MDST samples (Fig. 4). In terms of agreement calculation, if the relative abundance of species i shifts in the same direction in both simulated and real pre-FMT and post-FMT samples of a given patient, we consider species i as having consistent abundance movement or “agreeing” between the two. For the recovery degree, μ = $\frac{x^{d}-x^{p}}{x^{d}-x^{h}}$, where $x^{h}$, $x^{d}$, and $x^{p}$ are the normalized abundances of target pathogens in healthy (in this case, we assume it is 0), diseased and post-MDST states. The raw data are available at: https://gitlab.com/treangenlab/bakdrive.

### 2.9 Statistical analysis

Bray-Curtis similarities and principal coordinate analysis (PCoA) is conducted using skbio.diversity and skbio.stats.ordination packages (The scikit-bio development team 2020). Statistical significance testing is decided by Wilcoxon rank-sum using the scipy.stats.ranksums package (Virtanen et al. 2020). The script is available at: https://gitlab.com/treangenlab/bakdrive.

## 3. Results

### 3.1 Simulated MDS transplantation in rCDI patients

In this experiment, we investigate the impact of the number of network layers on the driver species prediction using simulated data. We first simulated 1000 pairs of diseased and healthy metagenomic samples following the protocol in Xiao et al. as described above. In this protocol, all the simulated samples share the same ecological network; the only difference between samples is the species abundance. After simulation, use Bakdrive to find driver species. Finally, we transplant the set of driver species to the 1000 diseased samples (excluding species already present in each one) and simulate species abundances change under the GLV model. To quantify the efficacy of MDST, we compute the *recovery degree*, μ, which represents how close the *C.difficile* abundance in the after-MDST state is to that of the healthy versus diseased states, expressed as a fraction of the difference between them (here μ=1 represents full recovery and μ=0 is none).

**Figure 2.**
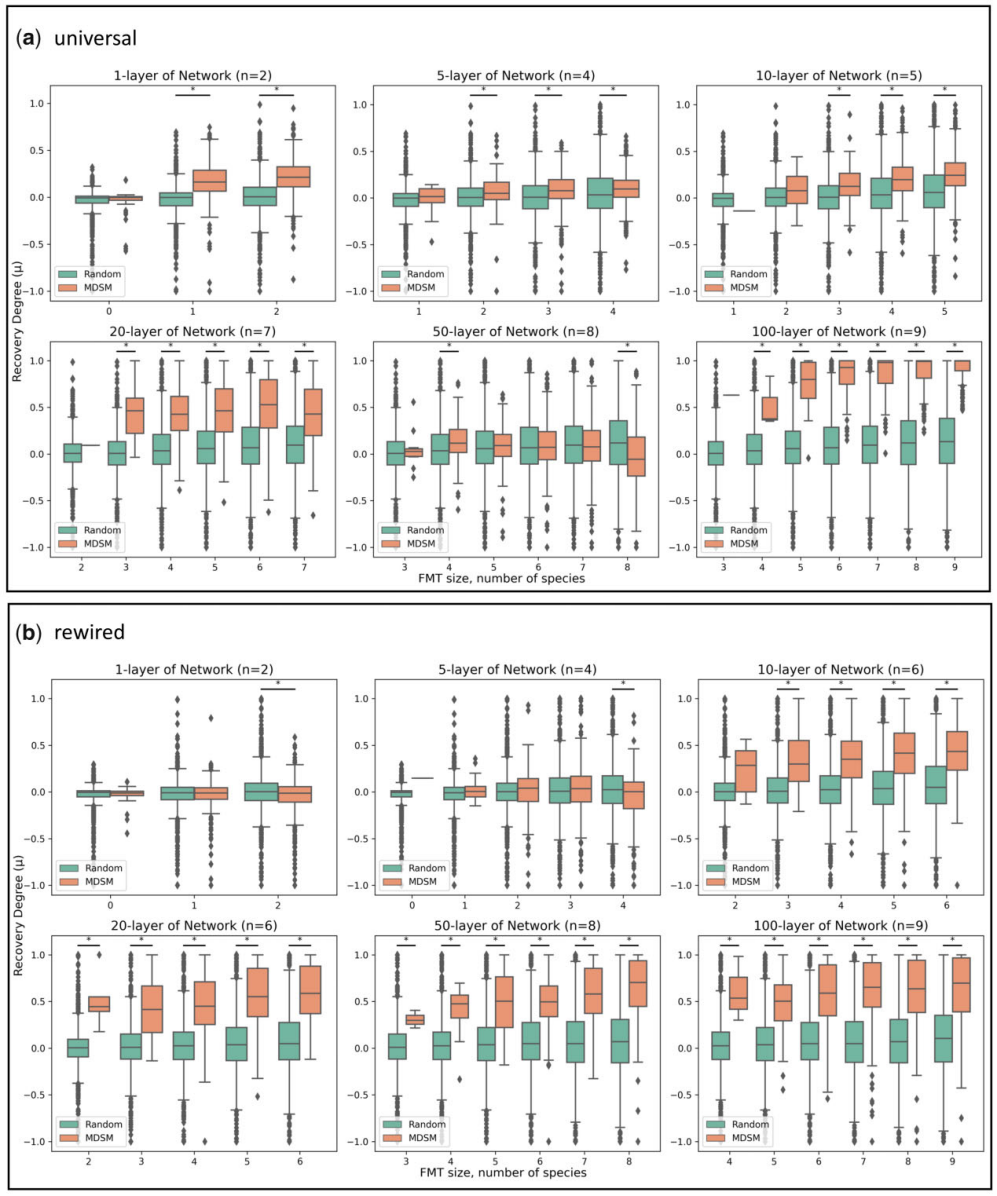
Efficacy of driver species transplantation with universal ecological networks. (a) Universal. (b) Rewired ecological networks. We simulate the driver species transplantation process with universal/rewired microbial dynamics. In each box plot, the *Y*-axis shows the recovery degree of *C.difficile* after the driver species transplantation over 1000 simulated engraftments. The *X*-axis represents the actual number of species introduced to the diseased samples, while n is the number of driver species identified from a given multilayer network, showing that there is an increase in efficacy as a greater number of the identified driver species are added. The orange boxes represent the recovery degree of *C.difficile* after colonizing driver species. The green boxes represent the recovery degree of *C.difficile* after introducing the same number of species, which are randomly selected from the global pool of 100 species. The significant differences of recovery degree by Wilcoxon rank-sum test are labeled by asterisks (*P < 0.05).

It is worth noting that, in this experiment, we did ***not*** use MICOM to infer ecological networks, as all the bacteria interactions were simulated based on the Xiao et al. protocol, with the simulated interactions provided to Bakdrive as an input. In this section, we focus on evaluating the ability of a driver species set identified by the MDSM algorithm (i.e. Bakdrive) to control microbiome state post-engraftment. Figure 2a shows the recovery degree for all 1000 samples after transplantation under various conditions. We start by computing the driver set using a subset of the 1000 samples, and the panels show results using subset sizes of 1, 5, 10, 20, 50, and 100. Sizes beyond 100 were tested but came with a substantial increase computation time and with little additional accuracy. The simulated transplantation excludes driver species that are already present in the diseased microbiota, so the actual number of transplanted species varies slightly across different samples. Additionally, for every condition, a control was run by randomly selecting an equivalent number of species to colonize. The control results are shown in green.

First, it is noteworthy that although the layer-count increases from 1 to 100, the size of the MDS goes only from 2 to 9. Second, the results clearly show that the MDS calculated by Bakdrive is effective at generating recovery by this measure. When *N* = 100 and MDS size is 9, substantially all simulated samples experience near-full recovery.

**Figure 3.**
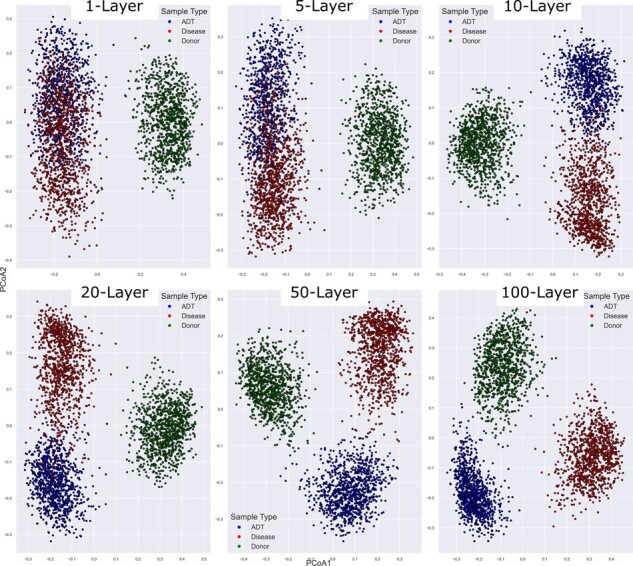
Principal coordinate analysis (PCoA) of simulated diseased (red), donor (green), and after driver species transplant (ADT) samples (blue). In each panel, we have 1000 simulations and thus 1000 samples of each type. The panels vary by the number of layers N (input samples) used to calculate the MDS (1, 5, 10, 25, 50, 100). PCoA was done using Bray-Curtis dissimilarity. As N increases the red and blue clusters (pre- and post-transplantation, respectively) become gradually more separated, showing that the MDS affects not just *C.difficile* abundance but the overall community.

Figure 3 shows the same phenomenon in a slightly different manner. Here again the panels represent the various sample sizes *N*, (or equivalently, *N*-layer networks within Bakdrive). Here, we have Bray-Curtis PCoA plots of the pre-MDST, post-MDST, and donor samples in each case. As *N* increases, the red and blue clusters (pre- and post-transplantation, respectively) become gradually more separated from one another. In other words, not only are the samples showing normalized *C.difficile* abundance, but the overall effect on the community is becoming more pronounced with more samples.

Next, we stress test our assumption about the interaction network. Specifically, we modify the bacteria interaction networks for each simulated sample using K rewiring (see Section 2), which preserves the degree of each node but alters the connections between them. This random perturbation is potentially draconian but is intended to create conditions where each sample has a unique bacterial interaction network and a different intrinsic growth rate vector, an important test for Bakdrive. Results are shown in Fig. 2b. Overall, the efficacies of driver species computed using rewired bacterial interaction networks are consistent with the results of universal interaction networks, though some tempering of the recovery degree is observed. Nonetheless, using the 100-layer rewired network driver species, nearly all post-MDST samples were at least 50% recovered.

### 3.2 Comparison with FMT-Induced changes in real patients

**Figure 4.**
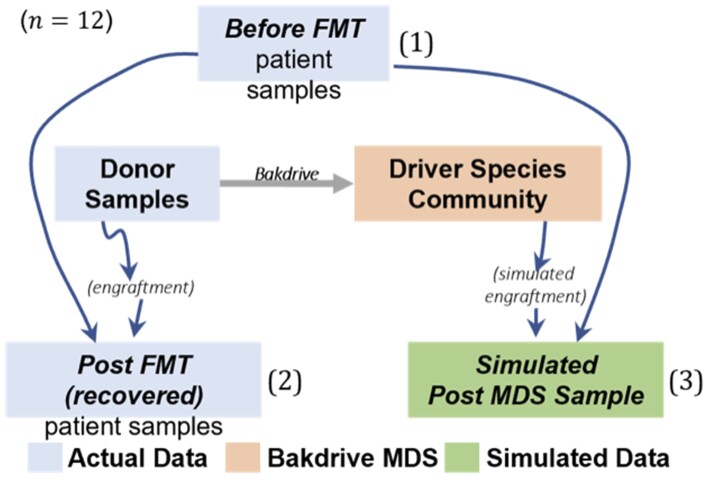
Experimental setup of rCDI actual FMT versus simulated MDS comparison. Microbiome compositions were taken from actual patient data where samples were available from (a) patient before FMT, (b) donor, and (c) patient after FMT (all shown in blue). 12 patients were considered where eventual recovery was attained (blue). The set of donor samples were used as input to Bakdrive to generate a mock driver species (MDS) community (orange). The actual pre-FMT samples were given a simulated engraftment of the MDS, with the resulting community development simulated (green). Of interest is whether the *actual* changes following FMT (i.e. (1) to (2)) resemble the *simulated* changes following MDS engraftment (i.e. (1) to (3)).

The previous experiments are encouraging but are strictly *in silico*, and thus depend on the simulation model appropriately reflecting empirical community maturation *in vivo*. To test this, we conducted an experiment intended to compare the changes induced by a simulated engraftment of a Bakdrive-computed MDS community to those induced by actual engraftment of FMT donor samples. The design of this experiment is shown visually in Fig. 4, and the comparison of interest is whether the changes from $\left(1\right)\to (2)$ are similar to those from $\left(1\right)\to (3)$. The appropriate metric to capture this comparison is non-obvious though; because of the inexact nature of the simulation framework, as well as general noise in estimating precise relative abundances from sequencing data, the “direction” of the abundance change for each microbe in the pre-FMT community was the operative measure of the changes. Agreement of the direction of change-in-abundance between the two processes was used as the measure of comparison. This metric has potential issues: one is that increases or decreases in relative abundance may be due to simple addition of a new population combined with the nature of compositional data. In this case, because the comparison is *after* a period of either simulated or actual community survival, we believe this effect is somewhat attenuated but worth noting.

**Figure 5.**
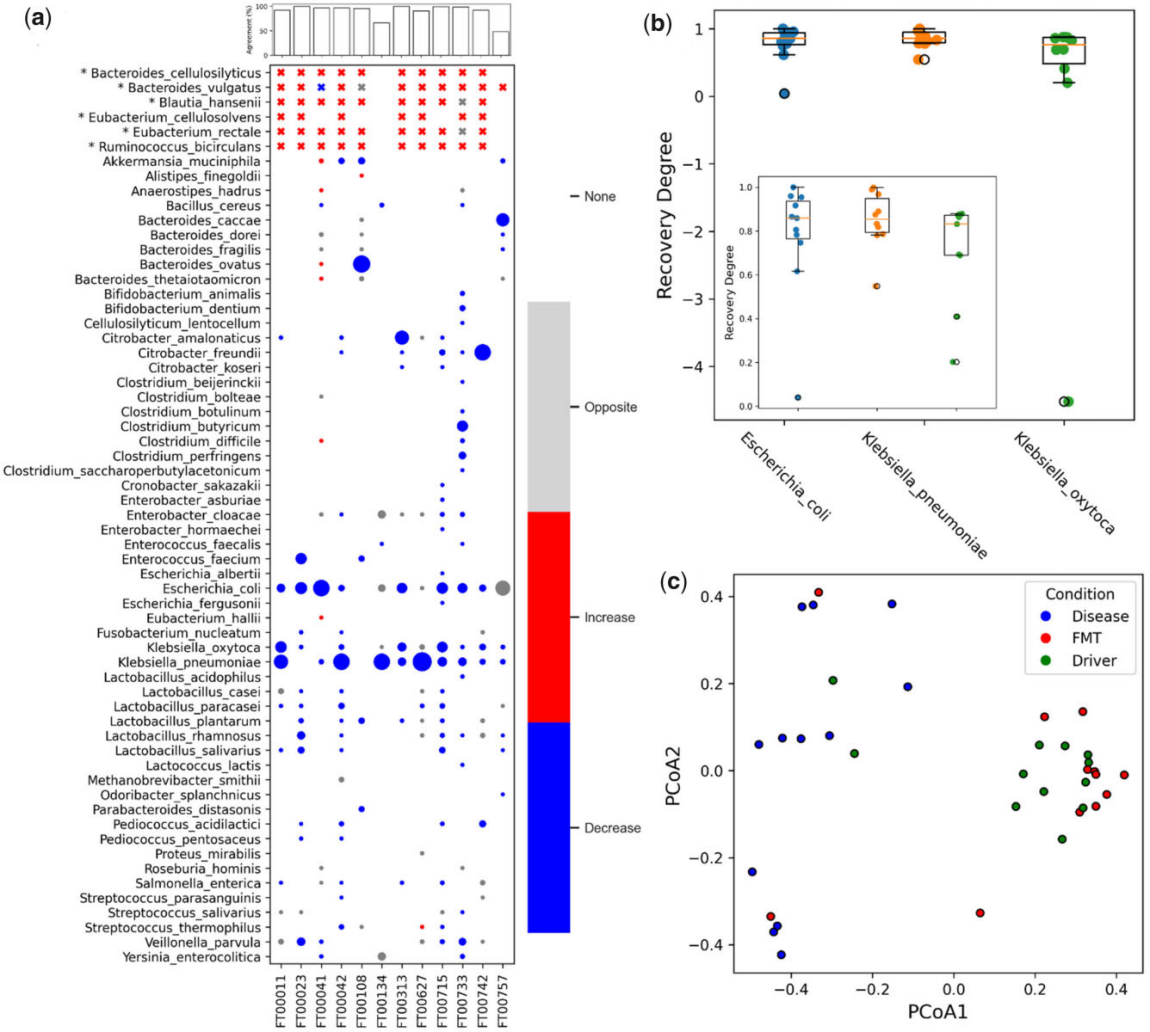
The consistency of species abundance changes between real and simulated samples. (a) Each column represents a patient sample. Each row represents a species that exists in 12 patient samples or belongs to driver species. “*” marks driver species. The driver species abundance changes are labeled as cross, as most driver species do not exist in diseased samples. The size of the bubble represents the percentage of species in patient samples before FMT. Gray shows species abundance changes in opposite directions between simulated and real dataset. Red/blue indicates a given species abundance increases/decreases after FMT in both simulated and real data respectively. White (“None”) indicates that the microbe was not present in the pre-FMT sample and thus no change was measured. In the upper bar plot, agreement quantifies the percentage of species in renormalized diseased samples (3) whose abundances shift in the same direction in both real and simulated data. (b) Recovery degree of pathogens. *x*-axis represents the target pathogens. *Y*-axis represents the recovery degree of target pathogens in each sample. We then zoom in the bar plot and only show samples with recovery degree above 0. (c) Principal coordinate analysis (PCoA) of real diseased, after FMT and after-MDST samples. Each sample is represented as a dot. There are a total of 36 samples from 12 patients. Bray-Curtis dissimilarity between samples are computed.

We detect a total of 8 driver species from the 26 donor samples (Fig. 5). Importantly, *Campylobacter jejuni* and *Streptococcus agalactiae* were identified as driver species in the donor samples and are known pathogens, and as a result were excluded from the driver-species for transplantation. This is a notable difference in the Bakdrive protocol for this experiment, but reflects sensible precautions likely to occur in actual practice.

The results are shown in Fig. 5a. Here the driver species are listed at top, followed by all other microbes in before-FMT samples. For each patient and each species, the table marker is colored based on whether abundance “increased” in both simulated and actual (red), decreased in both (blue), or changed in opposite directions (grey). The table is overwhelmingly blue and red, indicating broad agreement. For each patient, the percentage of species in agreement is also shown in a bar chart at the top, and is above 90% in all but two patients. A null hypothesis for comparison in this case would be the percentage of species that decrease on average in the *in vivo* samples which is 58.5%. The overall mean agreement is 88.8% [95% confidence interval (CI) 79.6–97.7%] and is statistically significant (Supplementary Table S2). This is a broad indication that the simulated recoveries generated by the MDST reflect the same mechanisms at work in FMT successes, a very encouraging result.

We additionally wanted to account for the possibility that the FMT and MDST changes would agree simply because resident pre-FMT microbes decrease in abundance whereas donor/MDS species increase. For comparison, we additionally simulated the MDST on the seven patients who failed to recover from a single FMT treatment (Supplementary Fig. S5). It shows that not all the resident species decrease in the FMT simulation. In these patients the mean agreement is slightly lower at 74.3%, but even with only 7 patients the % agreement is significantly different from 50% (CI*:* 54.1–94.5%). The abundances of *Escherichia coli* increase in most of the samples. Since *E.coli* is considered as pathogenic species in rCDI, it implies that the driver species is not “panacea”. Different types of patients may require different sets of probiotic treatments.

In Fig. 5b, we borrow the concept of recovery degree from earlier, but here we apply it to three pathogens from the genera *Klebisella* and *Escherichia*, known to be dominant in rCDI patients, (Park et al. 2019). We calculate the recovery degrees of *E.coli*, *Klebsiella oxytoca*, and *Klebsiella pneumoniae*, which average 0.78, 0.63, 0.85, respectively. This is yet another metric in which the *in silico* engraftment of the driver species closely reflects the recovery progression of *in vivo* FMT recipients.

Figure 5c contains Bray-Curtis PCoA plots of the disease-state, the post-FMT (*in vivo*), and post-MDST (*in silico*) samples from 12 patients. The plot makes it visually clear that overwhelmingly the post-FMT and post-MDST samples cluster closely together (at right), whereas the disease samples are separated and clustered together (at left). Again, this indicates that the post-engraftment progression of the simulated patients getting the community of driver species only closely reflects the progression of actual patients given the much more complex donor community in an FMT. Thus the *in silico* model gives every indication the Bakdrive MDS engraftment is indeed a functional facsimile of FMT.

## 4. Discussion

Recent research has shown the limitations of interaction networks based on co-occurrence patterns, and have suggested ecological networks calculated from biochemical reactions and metabolomics profiling (Menon et al. 2018; Hirano and Takemoto 2019; Frioux et al. 2020). This has led to recent focus on interaction networks through the lens of biochemical reactions and controllability analysis (Coyte et al. 2015) and efforts to try regulating microbial communities through controllability analysis (Loehle 2006; Angulo et al. 2019). Bakdrive belongs in this same genre and draws from control theory in identifying driver species, though it differs from others in some important specifics. The major ones have been noted above: (i**)** that it does not require a known interaction network, (ii**)** it uses networks that vary per sample. But an important difference from the most recent method in Xiao et al. is that the method presented there, in one step, uses reduction in *C.difficile* abundance as essentially a supervision criteria to select the driver-species. That makes it necessarily specific to rCDI, whereas Bakdrive is phenotype-agnostic and could possibly be applicable to additional clinical indications, such as metagenome-related diseases driven by unknown multiple pathogens. For rCDI specifically, a benchmark comparison of the two methods shows they give qualitatively similar results (Supplementary Fig. S3), but Bakdrive’s general applicability is an advantage.

It has long been known that FMT procedures carry health risks for the recipient, a major category of which is systemic infection from pathogens acquired from the donor. An outbreak of infection from *E.coli* in March 2020 led to a change in screening procedures and an FDA advisory. In that context, the observation that driver species identified from the 12 FMT donor samples included two out of eight that were known pathogens is both alarming and, perhaps, could have been expected. In fact, the one sample with low agreement was due primarily to a sharp increase in actual abundance of *E.coli* following the FMT. That the driver species include “other” known pathogens reaffirms that FMT, for all its success, is far from a panacea. It also reaffirms the potential value in using Bakdrive or another contribution from control theory to identify a simpler set of microbes, which can be monitored for safety and introduced to the recipients with the same beneficial effect.

### 4.1 Limitations and future directions

One important observation from this study is that there is no “one” optimal set of driver species for each phenotype. Driver species identification through this algorithm is influenced by many factors, including selection of input samples, accuracy of taxonomic classification, availability of species-level, genome-wide metabolic models, et cetera. For the sake of testing this, the simulation experiment described above was repeated twice with different random seeds, and even at *N* = 100 layers, the set of driver species identified differs non-trivially between replicates (Supplementary Table S6). That does not imply that the results themselves are unreliable, but rather that the identities of the particular species may not be as important as properties of the collective relationships to the rest of the community. Also, this result is consistent with Xiao et al. conclusion that there may not be a generic probiotic cocktail that works for all patients. But future work will ideally build on our understanding of how two largely non-overlapping sets of driver species can operate equally effectively.

Bakdrive additionally pairs two methods designed for completely unrelated contexts and applications (MICOM and MDSM), and has features which are suboptimal for this particular application. For one, Bakdrive does not take the directionality and strength of ecological networks into consideration. Also, the multilayer MDS problem is NP-hard, so the MDSM algorithm itself is necessarily a heuristic and may have room for improvement on its own terms, and possibly additionally so in the context of this novel use-case. Finally, although MICOM offers advancement in the inference of bacterial interaction it is environment-specific (the human gut) and may also be improvable. Bakdrive can readily integrate additional ecological inference software in the future. Due to the limitations of MICOM, our Bakdrive software also provides a function in which users can input ecological networks themselves to identify driver species.

Finally, an important next step is identifying when a set of driver species are “compatible” in the sense that they can be combined in a form of probiotic “cocktail” for dosing to a patient. Xiao et al. suggests that the probiotic cocktail should exclude species already present in the recipient’s microbiome, and there may not be one perfect set of microbes that works for all patients. It may indicate that the probiotic cocktail selection and formulation is a more involved process.

## 5. Conclusion

As a general matter, methods designed to identify “core” or “driver” species within a community do so despite that term actually being only vaguely defined: typically it means species that are in some sense important to stability or that have notably strong interactions with other community members. In the context of rCDI though, the risks associated with FMT imply a more direct definition: a small subset of species whose “engraftment into a new environment leads to essentially the same outcomes as would the engraftment of the entire parent community.” In other words, in terms of community transfer the subset serves as a reasonable facsimile of the whole parent population. Bakdrive is a novel combination of two methods from separate disciplines which, together, make up an algorithm for identifying such a set when given as input a representative sample of different community profiles for a particular environment.

What sets Bakdrive apart from other methods is specifically the structure of this input data: information is gained by the variety of examples included, and no separate analysis of interaction networks is needed. Our experiments suggest that as a result, it is quite effective at identifying such a proxy subset, and further that there is nothing limiting its use to rCDI or even the human gut environment (except possibly MICOM, although it could be extended with some effort to other environments). In theory then, if hypothetically (i**)** Bakdrive driver-subsets turn out to be just as effective in practice as they appear to be in simulation, and (ii**)** that this is true for a wide range of target environments, it has the potential to be a very powerful tool for engineering artificial microbiomes for a desired function. That is obviously a highly optimistic hypothetical, of course, but Bakdrive nonetheless represents a potential step forward in this area and should lead to some interesting experiments with transfer of community function in unrelated environments.

## Supplementary Material

btad236_Supplementary_Data

## Data Availability

The data underlying this article are available in the article and in its online supplementary material.
