# Supplementary material for: Bakdrive: identifying a minimum set of bacterial species driving interactions across multiple microbial communities
Source: Bioinformatics. 2023 Jun 30;39(Suppl 1):i47–56. doi: 10.1093/bioinformatics/btad236 (PMC10311314; doi:10.1093/bioinformatics/btad236)
Supplement: btad236_Supplementary_Data [file btad236_supplementary_data.pdf]

# Bakdrive: Identifying a Minimum Set of Bacterial Species Driving Interactions across Multiple Microbial Communities

## Supplementary Figures and Tables

Qi Wang[1], Michael Nute[3], Todd J. Treangen[2*]

[1] Systems, Synthetic, and Physical Biology (SSPB) Graduate Program, Rice University, Houston, Texas, USA

[2] Department of Computer Science, Rice University, Houston, TX, USA

[3] Anvil Diagnostics, Southborough, MA, USA

*To whom correspondence should be addressed.

# Expanded Discussion of Bakdrive Algorithm Details

*Nota Bene: This section includes a longer and more in-depth discussion of the Bakdrive pipeline and algorithm. In particular some of the content here is drawn from earlier drafts of the manuscript when the same space restrictions were not in place, and thus with a longer methods section in the main paper. However, not all of the content from the previous draft was cut, so this section may duplicate some parts of the main paper in conjunction with additional discussion.*

## A. Expanded Discussion of "Recovery Degree" Metric in Simulations

In the manuscript we briefly described how the "Recovery Degree" was calculated as it was used to evaluate the "success" of the FMT in our simulated study. The formula for the Recovery Degree is given here as $\mu$ and with some additional clarifying detail:

$$\mu = \frac{x^d - x^p}{x^d - x^h}, \qquad \text{where} \begin{cases} x^d \text{ is the percentage in the diseased state} \\ x^h \text{ is the percentage in the healthy state} \\ x^p \text{ is the percentage in the post-FMT state} \end{cases}$$

This metric is intended to capture the degree to which the microbiome after FMT has returned to the healthy state, specifically the fraction of the difference between *a priori* diseased/healthy concentrations that has been recapitulated in the post-FMT community.

## B. MDSM algorithm

Before applying the MDSM algorithm, we need to convert a set of weighted, directed bacteria interaction networks into a multilayer network, where each layer is an undirected and unweighted bacterial interaction network $G_k(V_k, E_k)$ of a metagenomic sample k.

The goal of the MDSM algorithm is to find a minimum set of driver species that are crucial in controlling a given metagenomic state. The metagenomic state is characterized by the multilayer network. The problem of finding driver nodes from a multilayer network is simplified as a binary integer linear programming (ILP) problem (Nacher and Akutsu, 2012):

$$\text{minimize } f(x) = \sum_{i=1}^{n} x_i$$
$$\text{Subject to } x_i + \sum_{j:\{vi,vj\}\in E_k}^{n} x_j \geq 1 \text{ for all } v_i \in V_k \text{ for all k = 1, …, N,}$$

where f(x) is the dominating set and $x_i$ is a binary variable of each node. $x_i$ = 1 while node i belongs to the dominating set f(x). n is the total number of nodes and N is the total number of layers of a given multilayer network. Driver species identification is achieved by Bakdrive *driver* module.

## C. Real rCDI and CD metagenomic data analysis

The rCDI metagenomic data includes 26 donor samples from 7 donors, 19 patient samples and their corresponding after-FMT samples. Among 19 patients, 12 of them have fully recovered after receiving a single dose of FMT, while the remaining 7 patients need to receive a 2nd dose of FMT. For the simplicity of this study, we focus on analyzing the FMT results of the 12 patients with single successful FMT. The raw sequencing of rCDI is available at BioProject PRJNA454892. The sequences are classified at species level using Kraken with the full database (Wood and Salzberg, 2014).For CD, the taxonomic classification results of the HMP2 whole genome sequencing pilot dataset were directly downloaded from the IBDMDB database https://ibdmdb.org/tunnel/public/summary.html (Franzosa et al., 2018; Lloyd-Price et al., 2019). In this study, species with relative abundances below 0.1% are removed.

To identify driver species from real metagenomic data, we first construct bacteria interaction networks of individual metagenomic samples. The newly developed software MICOM provides a function of inferring bacteria interactions and species growth rate through flux balance analysis (FBA) (Diener et al., 2020). Before FBA, MICOM matches species in each sample with their genome-scale metabolic models by name. In this work, `AGORA_1_03_With_Mucins` is used as the model database, which contains 818 reconstructed models (Magnúsdóttir et al., 2017). If a species has multiple strains' metabolic models in the database, we will randomly pick one as the representative model of the species. While conducting FBA, western diet is used as the default medium for growth simulation.

To infer bacteria interactions, MICOM conducts *in silico* knockout experiments and computes relative growth rate interactions between two species, denoted as $r_{ij}$. By knocking out species $i$, the relative growth rate interaction of species $i$ on $j$ ($r_{ij}$) is calculated. $r_{ij}$ is defined as:

$$r_{ij} = \frac{knockout\ growth\ rate\ j - original\ growth\ rate\ j}{original\ growth\ rate\ j}\ i \neq j \text{ , where } r_{ii} = -1$$

To obtain an interaction matrix for the following FMT process simulation, we need to convert $r_{ij}$ into the interaction strength of species $i$ on $j$. The meaning of interaction strengths is not constant among ecological literatures (Laska and Timothy Wootton, 1998). In this paper, interaction strength, denoted as $a_{ij}$, is defined as competition coefficient, which measures interspecific competition relative to intraspecific competition, $a_{ij} = \frac{r_{ij}}{r_{ii}}$. By this definition, the interaction strength $a_{ij}$ is negative (positive) while species $i$ have a negative (positive) impact on species $j$ respectively. This step is conducted by Bakdrive *interaction* module.

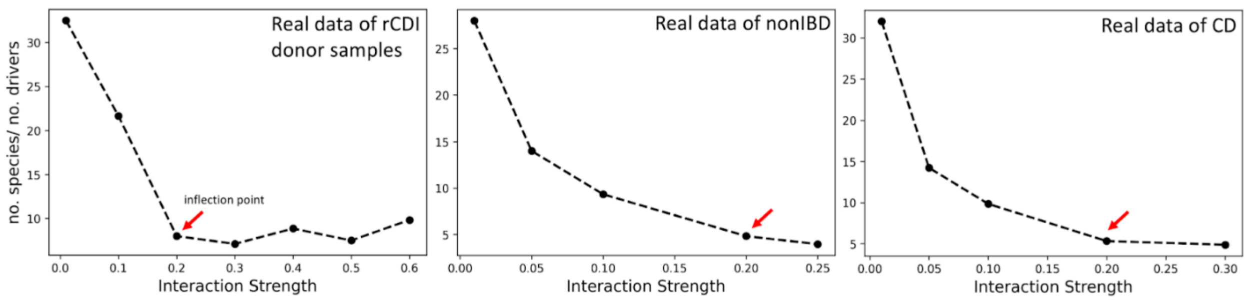


**Figure S1. Interaction strength threshold selection.** X-axis is the absolute value of interaction strength threshold. The edges with the interaction strength below the threshold are removed from ecological graphs. Y-axis represents **α** = total number of speciesnumber of drivers. Based on empirical experiments, the interaction strength threshold at the inflection point gives the best performance.

**Table S2. Confidence interval calculations for %-agreement metrics in Figures 5, S4 and S5**. All intervals are computed for 95% confidence using a normal approximation. Figure 5 contains patients who recovered following FMT while Figure S5 contains those who did not. Figure S4 includes the same patients as Figure 5, but using a network computed differently.

| | ***Figure 5*** | | ***Figure S5*** | | ***Figure 5,S5 (comb.)*** | | ***Figure S4*** | |
|---|---|---|---|---|---|---|---|---|
| | **Patient** | **Agree %** | **Patient** | **Agree %** | **Patient** | **Agree %** | **Patient** | **Agree %** |
| | FT00134 | 66.2 | FT00815 | 92.6 | FT00134 | 66.2 | FT00134 | 77.5 |
| | FT00023 | 99.8 | FT00387 | 75.1 | FT00023 | 99.8 | FT00023 | 97.1 |
| | FT00011 | 91.7 | FT00510 | 84.4 | FT00011 | 91.7 | FT00011 | 69.4 |
| | FT00757 | 47.9 | FT00642 | 73.2 | FT00757 | 47.9 | FT00757 | 45.8 |
| | FT00313 | 99.6 | FT00062 | 96.6 | FT00313 | 99.6 | FT00313 | 99.4 |
| | FT00041 | 96.6 | FT00602 | 82.5 | FT00041 | 96.6 | FT00041 | 95.3 |
| | FT00042 | 96.5 | FT00444 | 15.5 | FT00042 | 96.5 | FT00042 | 90.9 |
| | FT00627 | 90.7 | | | FT00627 | 90.7 | FT00627 | 94.5 |
| | FT00733 | 91.7 | | | FT00733 | 91.7 | FT00742 | 91.6 |
| | FT00108 | 98.3 | | | FT00108 | 98.3 | FT00733 | 98.1 |
| | FT00715 | 95.4 | | | FT00715 | 95.4 | FT00108 | 94.7 |
| | FT00742 | 91.7 | | | FT00742 | 91.7 | FT00715 | 97.7 |
| | | | | | FT00815 | 92.6 | | |
| | | | | | FT00387 | 75.1 | | |
| | | | | | FT00510 | 84.4 | | |
| | | | | | FT00642 | 73.2 | | |
| | | | | | FT00062 | 96.6 | | |
| | | | | | FT00602 | 82.5 | | |
| | | | | | FT00444 | 15.5 | | |
| (a) | Mean | 88.8 | | 74.3 | | 83.5 | | 87.7 |
| (b) | Sample S.D. | 15.7 | | 27.3 | | 21.2 | | 15.3 |
| (c) | Count | 12 | | 7 | | 19 | | 12 |
| (d) | Lower C.I. | 79.6 | | 54.1 | | 73.7 | | 78.8 |
| (e) | Upper C.I. | 97.7 | | 94.5 | | 93.0 | | 96.3 |

***Confidence Interval Calcs:***

(d) = (a) - 1.96 x [ (b) / sqrt((c)) ]

(e) = (a) + 1.96 x [ (b) / sqrt((c)) ]

# Comparison between *Xiao et al* and Bakdrive

To further evaluate the efficacy of driver species in decolonizing *C. difficile*, we benchmark Bakdrive with the analysis pipeline proposed in Xiao et al (Xiao et al., 2020). In this study, the authors utilizes a time-series analysis inferred ecological network as initial network (Bucci et al. 2016) and simulate two rCDI metagenomics samples following the GLV model. The initial ecological network contains a total of 14 species, including GnotoComplex microflora, a mixture of human commensal bacteria and *C. difficile* (Figure S3a, column 1). The simulated rCDI metagenomes have subsets of the 14 species and high abundances of *C. difficile* (Figure S3a Column 2&4). In order to compare the performance between Bakdrive and the Xiao *et. al.* pipeline, we reproduced the two rCDI metagenomes. Additionally, we simulated 50 donor samples from the initial ecological network and identified 5 driver species from the donor samples using Bakdrive. For each rCDI sample, we colonize Xiao's personalized probiotics (Figure S3a, columns 3 and 5) and Bakdrive's driver species (Figure S3a, column 6), excluding species that exist in the rCDI microbiota, respectively. Compared to Bakdrive, the probiotics proposed by Xiao *et al* perform better in the first experiment, but worse in the second one, but both appear to contribute to substantial recovery (Figure S3b).

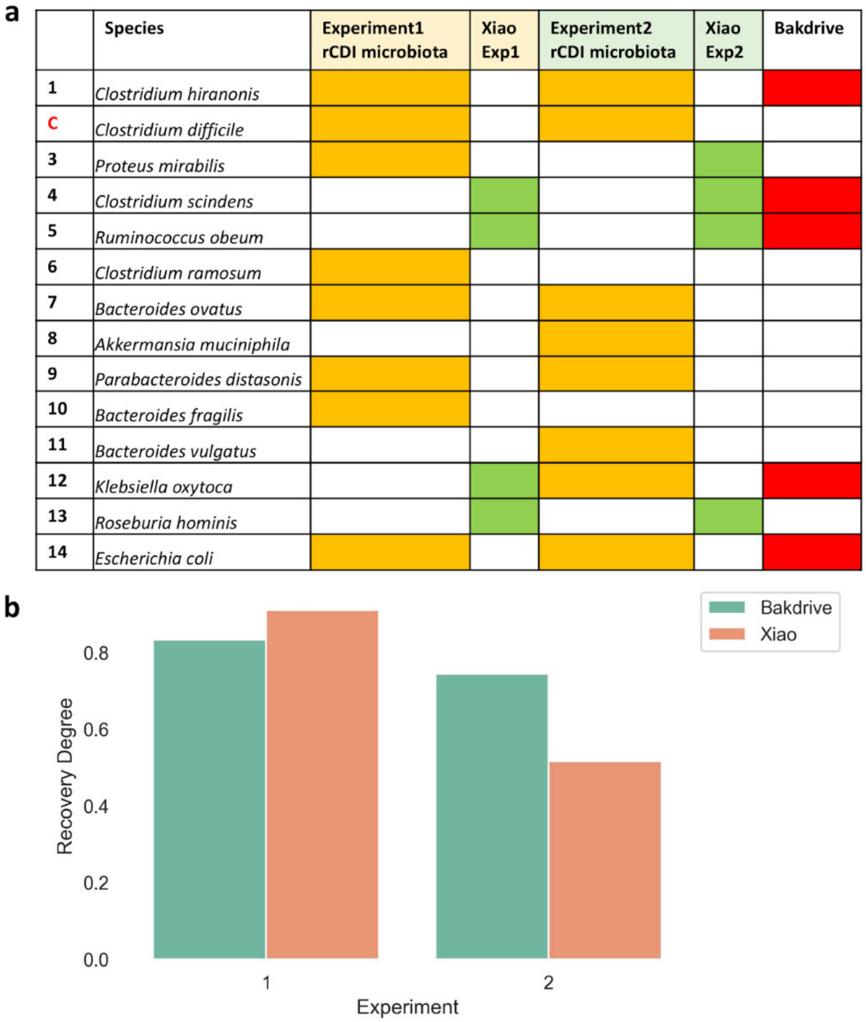


**Figure S3 Benchmarking recovery degree of rCDI after probiotics transplantation. a)** Species in simulated rCDI microbiota and driver species. Column 1: Species in GnotoComplex microflora and *C. difficile*. Column 2 & 4: species present in simulated rCDI microbiota in *Xiao et al* study. Column 3 & 5: beneficial species proposed by *Xiao et al* study. Column 6: driver species identified by Bakdrive. **b)** Recovery degree after *Xiao et al* or Bakdrive's probiotic cocktails introduction.

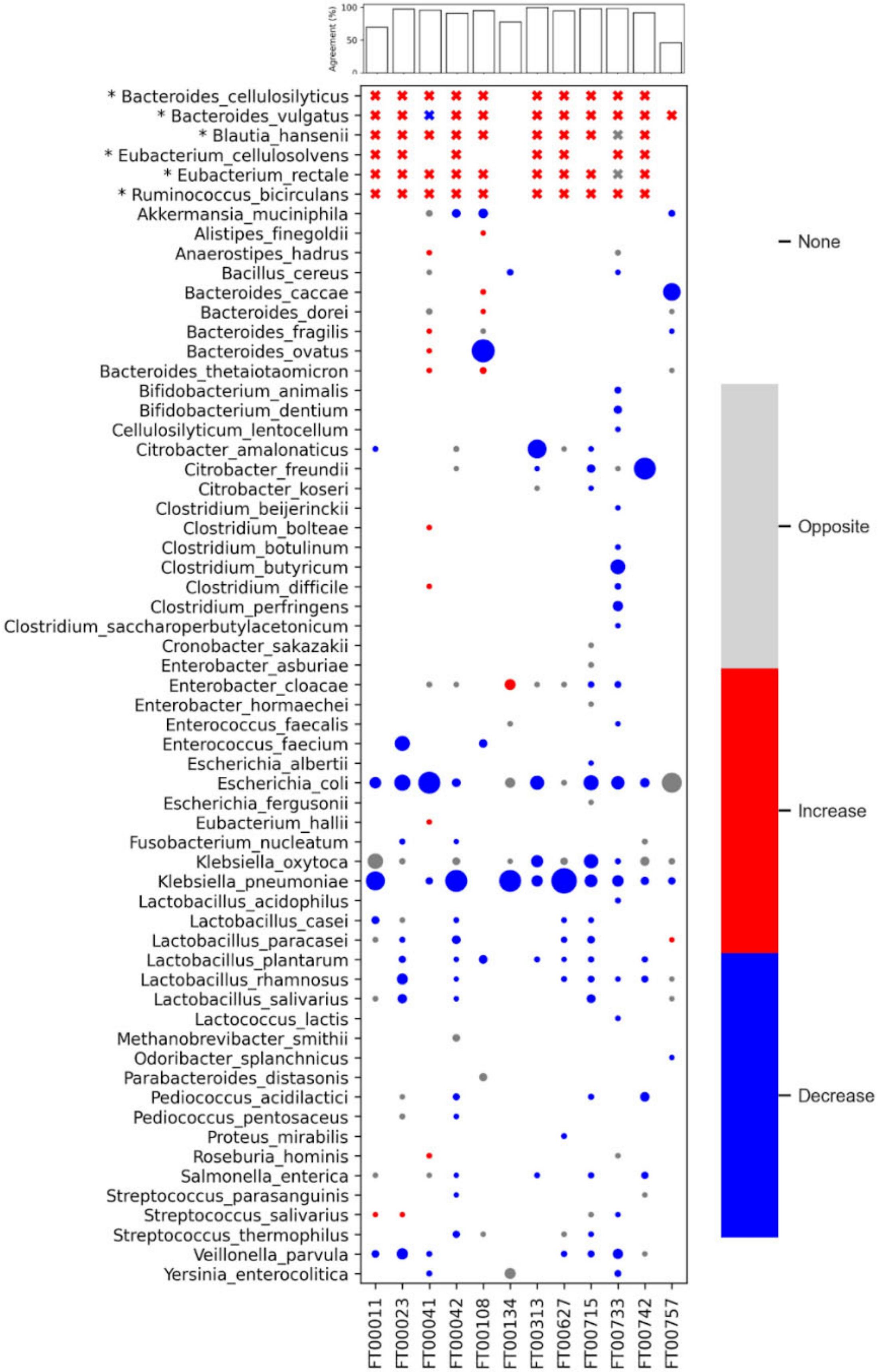


**Figure S4. The consistency of species abundance changes between real and simulated samples using normalized interaction matrices**. The interaction matrix is calculated by taking individual abundances into consideration.

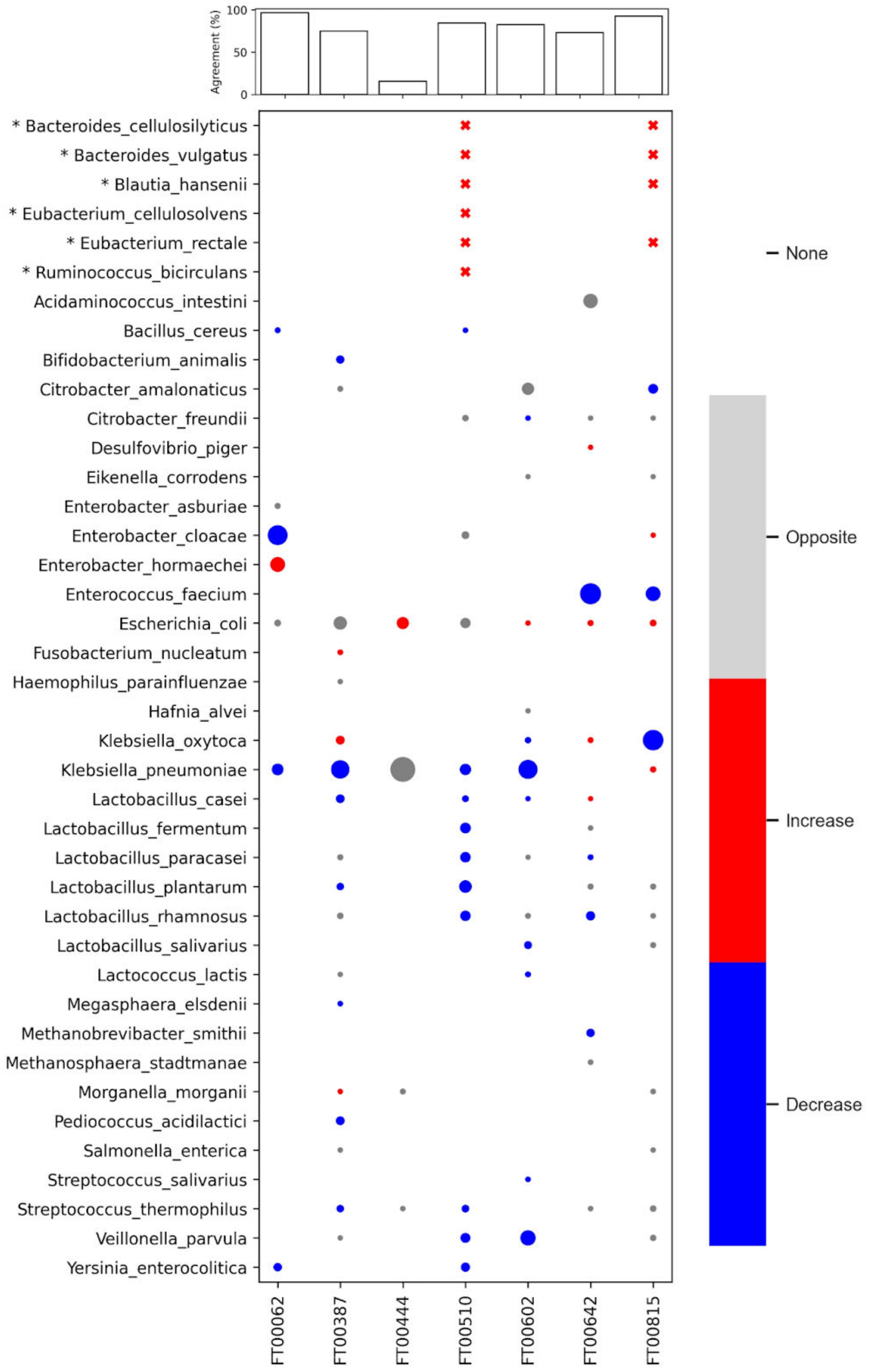


**Figure S5. The consistency of species abundance changes between real and simulated samples of patients who failed to recover from the first FMT treatment**. It shows that Escherichia coli increases in most of the patients. It implies that the set of identified driver species is not “panacea”.

**Table S6. Driver nodes of simulated data with universal ecological networks.** The main simulation experiments for our manuscript were performed three separate times with each simulation used as the precursor for a Bakdrive computation of the set of minimum driver species (MDS). As noted in the Limitations and Future Directions section, the driver set is not necessarily unique and the particular set of driver species notably varied across the three replicates, although the *size* of the driver species set was consistent. Regardless of the particular set used, the impact of engraftment in simulation was substantially the same (Repeat 3 was the specific replicate shown in the text). The table below shows the particular driver species selected for each experiment across all three replicates. **Bold** indicates driver nodes which appear in all repeats of 100-layer networks.

| No. of layers | No. of drivers | Driver Nodes Id | | |
|---|---|---|---|---|
| | | Repeat1 | Repeat2 | Repeat3 |
| 1 | 2 | 56,92 | 60,68 | 86,92 |
| 5 | 4 | 4,33,62,63 | 60,64,68,79 | 52,53,63,76 |
| 10 | 5 | 22,61,71,73,82 | 26,36,38,60,68 | 7,53,76,92,99 |
| 20 | 5-7 | 14,25,27,60,68,87,94 | 22,26,54,93,94 | 53,58,76,82,93,99 |
| 50 | 8-9 | 27,46,51,50,60,62,68,73,82 | 3,30,46,54,62,63,92,94 | 7,14,15,22,27,50,61,92 |
| 100 | 9 | 14,**22**,25,3,30,54,80,**92**,94 | 7,**22**,33,46,60,69,**92**,93,94 | 14,**22**,25,27,46,54,64,84,**92** |
